# Efficacy of acupuncture in the management of post-apoplectic aphasia: a systematic review and meta-analysis of randomized controlled trials

**DOI:** 10.1186/s12906-019-2687-1

**Published:** 2019-10-25

**Authors:** He-yong Tang, Wei Tang, Feng Yang, Wei-wei Wu, Guo-ming Shen

**Affiliations:** 1 Department of Acupuncture, Anhui University of Chinese Medicine, Hefei, 230012 Anhui China; 2 Department of Graduate, Fudan University, Shanghai, 200433 China; 3 Department of Rehabilitation, Second Affiliated Hospital of Anhui University of Chinese Medicine, Hefei, 230012 Anhui China

**Keywords:** Acupuncture, Postapoplectic aphasia, Meta-analysis, Randomized controlled trail

## Abstract

**Background:**

Aim of this study was to evaluate the effectiveness of scalp, tongue, and Jin’s 3-needle acupuncture for the improvement of postapoplectic aphasia.

**Method:**

PubMed, Cochrane, Embase databases were searched using index words to identify qualifying randomized controlled trials (RCTs). Meta-analyses of odds ratios (OR) or standardized mean differences (SMD) were performed to evaluate the outcomes between investigational (scalp / tongue / Jin’s 3-needle acupuncture) and control (traditional acupuncture; TA and/or rehabilitation training; RT) groups.

**Results:**

Thirty-two RCTs (1310 participants in investigational group and 1270 in control group) were included. Compared to TA, (OR 3.05 [95% CI: 1.77, 5.28]; *p*<0.00001), tongue acupuncture (OR 3.49 [1.99, 6.11]; *p*<0.00001), and Jin’s 3-needle therapy (OR 2.47 [1.10, 5.53]; *p* = 0.03) had significantly better total effective rate. Compared to RT, scalp acupuncture (OR 4.24 [95% CI: 1.68, 10.74]; *p* = 0.002) and scalp acupuncture with tongue acupuncture (OR 7.36 [3.33, 16.23]; *p*<0.00001) had significantly better total effective rate. In comparison with TA/RT, scalp acupuncture, tongue acupuncture, scalp acupuncture with tongue acupuncture, and Jin’s three-needling significantly improved ABC, oral expression, comprehension, writing and reading scores.

**Conclusion:**

As treatments to postapoplectic aphasia, scalp / tongue acupuncture and Jin’s Three-needling are found better than TA and/or RT in yielding total effective rate and improving ABC, oral expression, comprehension, reading and writing scores.

## Background

Stroke is a major cause of disability and mortality worldwide [1–3]. In 2013, stroke was the second most common cause of death (11.8% of all deaths) worldwide when the incidence of stroke was 3.28 million in women and 3.62 million in men [4]. Worldwide, stroke is the second leading cause of death and third biggest source of disability. Incidence of stroke has doubled in low-income countries in the last 4 decades whereas it has decreased by 42% in high-income countries. Stroke-related mortality is also much higher in low-income countries [5]. According to the American Heart Association, approximately 600,000 new individuals suffer stroke and 185,000 suffer recurrence of stroke each year. Healthcare and societal costs have increased from $53.6 billion to $68.9 billion from 2004 to 2009 (22% increase) [6].

Besides aging population, the contributory factors include work-related stress and dietary and lifestyle patterns due to which stroke morbidity is increasing even in younger individuals. Modifiable risk factors for the incidence of stroke include high blood pressure, diabetes, tobacco smoking, hyperlipidemia, obesity, unhealthy diet, and physical inactivity [7]. Besides significant mortality, post-stroke disability causes a great burden to sufferers and caregivers.

Acupuncture is a prominent treatment in traditional Chinese medicine for over 3000 years for several diseases including post-stroke recovery. Among important methods, scalp acupuncture is carried out based on the functionality of brain areas to stimulate different scalp zones with needles which improves the reflexively of certain nerves [8]. Tongue acupuncture involves insertion of acupuncture needle into the acupoints on tongue followed by twisting it usually for multiple times [9]. In Jin’s 3 needles acupuncture, 3 needles are inserted on strategic acupoints depending on the target symptom such as slurred speech, difficulty swallowing, spastic paralysis, aphasia, etc. [10].

Previously, Zhang et al. [11] and Wu et al. [12] conducted meta-analyses of studies which performed acupuncture for poststroke rehabilitation and found that acupuncture provided significant benefits in stroke rehabilitation. The Ottawa Panel evidence-based clinical practice guidelines also identify acupuncture as one of the treatment options for post-stroke rehabilitation [13]. The purpose of this study was to comprehensively review the randomized controlled trials (RCTs) which evaluated the effectiveness of scalp acupuncture, tongue acupuncture, and Jin’s 3-neddeling by comparing with traditional acupuncture or rehabilitation training for participants with post-stroke aphasia and to perform meta-analyses of important endpoints in order to gain clinically meaningful synthesis of present day evidence.

## Methods

### Search strategy

Literature search was conducted in PubMed, Cochrane and Embase databases for RCTs which evaluated the effectiveness of acupuncture for the rehabilitation of postapoplectic aphasia. Important keywords used for the search included acupuncture, scalp, tongue, intradermal, acupoint, apoplexy, stroke, cerebral apoplexy, cerebral infarction, cerebral hemorrhage, aphasia, cerebral infarction, randomized controlled trial, and RCT. Full literature search strategy is given as Additional file 1: Appendix S1 (online Supporting information file). Reference lists of important research and review articles were also screened. The search was completed by October 2018. Two reviewers conducted literature survey independently and then unified their outputs. For the screening and selection of studies, help of an additional researcher was sought who participated in eligibility criteria formulation earlier.

### Inclusion and exclusion criteria

A study was included if (i) was RCT, (ii) subjects had post-apoplectic aphasia without further severe disease diagnosed with WHO criteria using appropriate radiological methods (iii) the interventions included scalp acupuncture, tongue acupuncture, or Jin’s 3-needling, (iv) had a control group with traditional acupuncture and/or rehabilitation training interventions, and (v) published in English or Chinese language.

A study was excluded if: (i) the outcomes were based on acupuncture or related therapies other than scalp acupuncture, tongue acupuncture, Jin’s 3-needling or traditional acupuncture, (ii) the interventions of either treatment or control group included medication, (iii) outcomes were reported in formats that could not be used in meta-analyses of odds ratio (OR) or standardized mean difference (SMD), or (iv) was systematic review, meta-analysis, theoretical research, expert commentary, conference report, economic analysis, or was a case report/study.

### Data extraction, quality assessment and data analysis

Demographic data including the name of author (study identity), publication year, age, gender sample size, study quality (Jadad score), interventions of the treatment and control groups, and outcome data including total effective rate, the Aphasia Battery of Chinese (ABC) score, and the scores of comprehension, oral expression, repetition, denomination, reading and writing tests were extracted from the individual studies and organized in datasheets. Data were extracted by 2 reviewers independently which were then unified and validated with the involvement of a third reviewer.

The Jadad checklist was employed to assess the quality of the included studies. All RCTs were evaluated on the basis of five items: (i) statement of randomization, (ii) use of double-blind methods, (iii) appropriateness of generating randomized sequence, (iv) details of withdrawals and dropouts and (v) description of double blinding method. Studies with a score of < 3 signified low-quality and a high bias risk.

Meta-analyses of SMD were performed using STATA software (version 10.0; Stata Corporation, Texas, USA). Outcome variables reported as means and standard deviations (the ABC scores and subscale scores of comprehension / oral expression / repetition / denomination / reading / writing) were used to calculate SMD between treatment and control groups and then inverse variance-weighted overall effect sizes were generated. Categorical outcomes (total effective rates) were used to perform meta-analyses of odds ratios (OR) using Review Manager (version 5.1.3, Cochrane) software.

The statistical heterogeneity of clinical trial data was assessed with Chi-squared and I^2^ indices. A Chi-squared *P* values of ≤0.05 and I^2^ > 50% was considered to show high heterogeneity of outcome data. In subgroup analysis, four subgroups were: (1) scalp acupuncture, (2) tongue acupuncture, (3) Jin’s 3-needling, and (4) scalp acupuncture with tongue acupuncture.

## Results

**Fig. 1:**
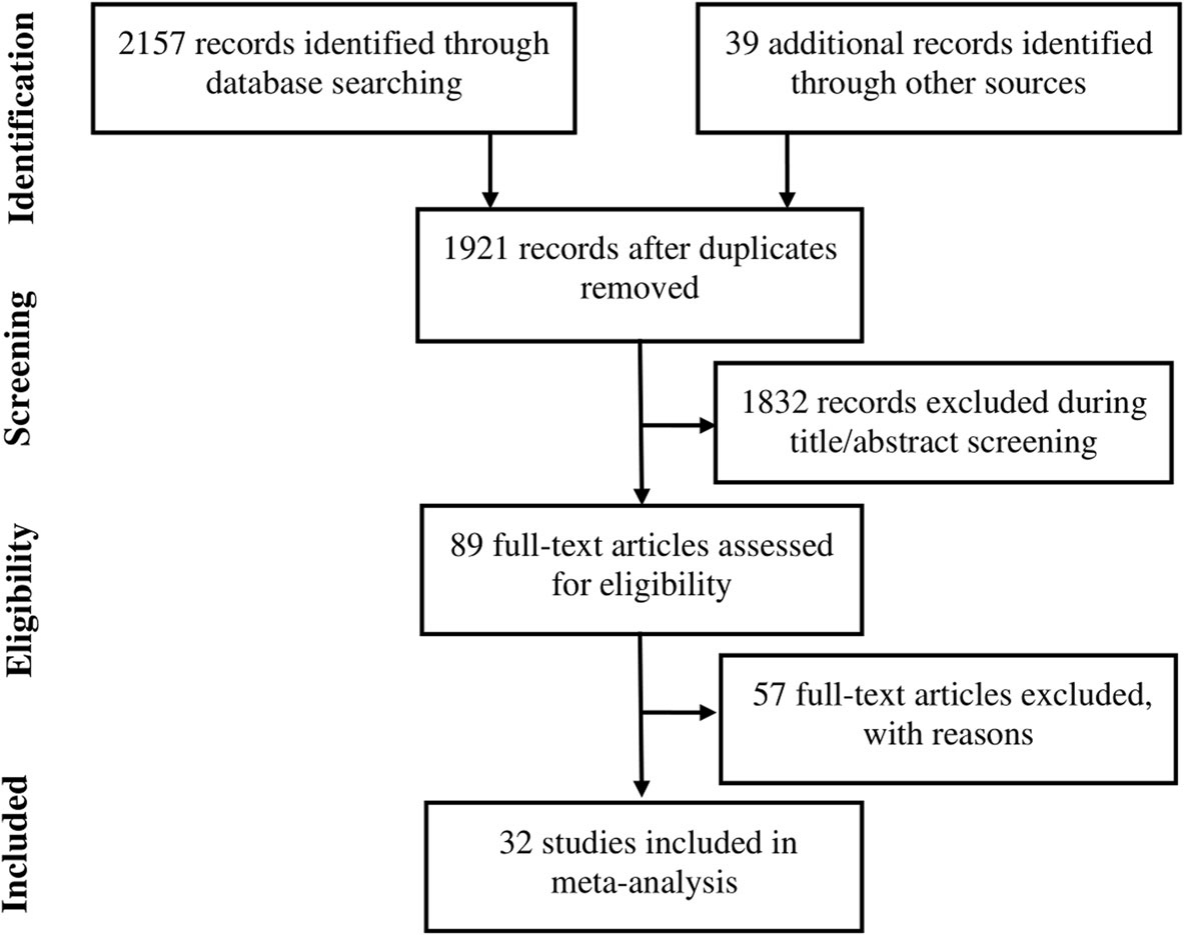
A flowchart of literature search and selection process

Database search identified 1921 publications of which 1832 were excluded after title and/or abstract screening and 89 were subjected to eligibility criteria application. After review of the complete manuscripts, 57 articles were further excluded because 18 were reporting non-RCT studies as 15 involved theoretical or economic research, 13 had unqualified interventions, and 11 and had no clinical outcomes. Finally, 32 RCTs were selected with 1310 participants in investigational group and 1270 in control group [14–45]. The screening and selection procedure is presented in Fig. 1. The study characteristics are presented in Additional file 1: Table S1.

**Fig. 2:**
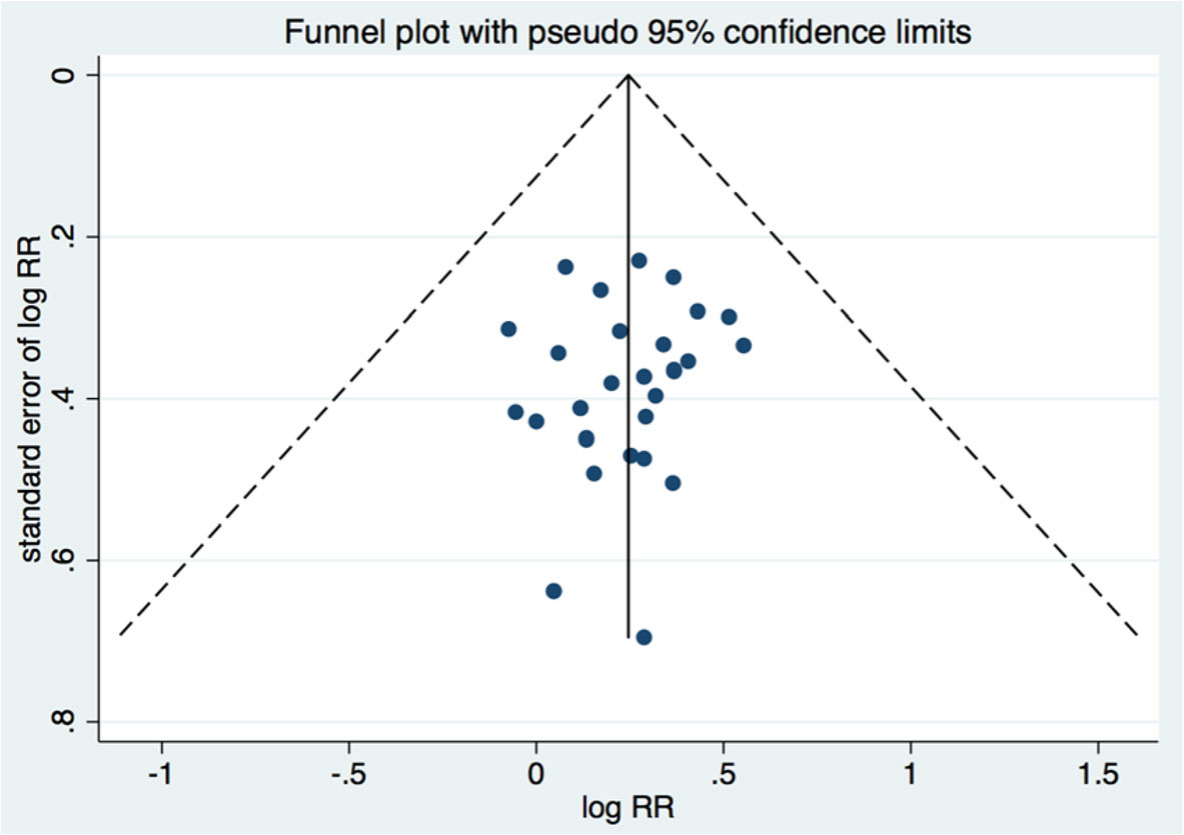
A funnel plot corresponding to the meta-analysis of total effective rate showing the absence of publication bias

The quality of the included studies was low, in general, as the overall Jadad score of included studies was 2.4. The funnel plot for total effective rate was symmetrical, depicting a lack of publication bias (Fig. 2). Begg’s and Mazumdar’s rank test (Z = 1.36, *p* = 0.173) and the Egger’s test (*p* = 0.362) also endorsed this finding.

### Total effective rate

**Fig. 3:**
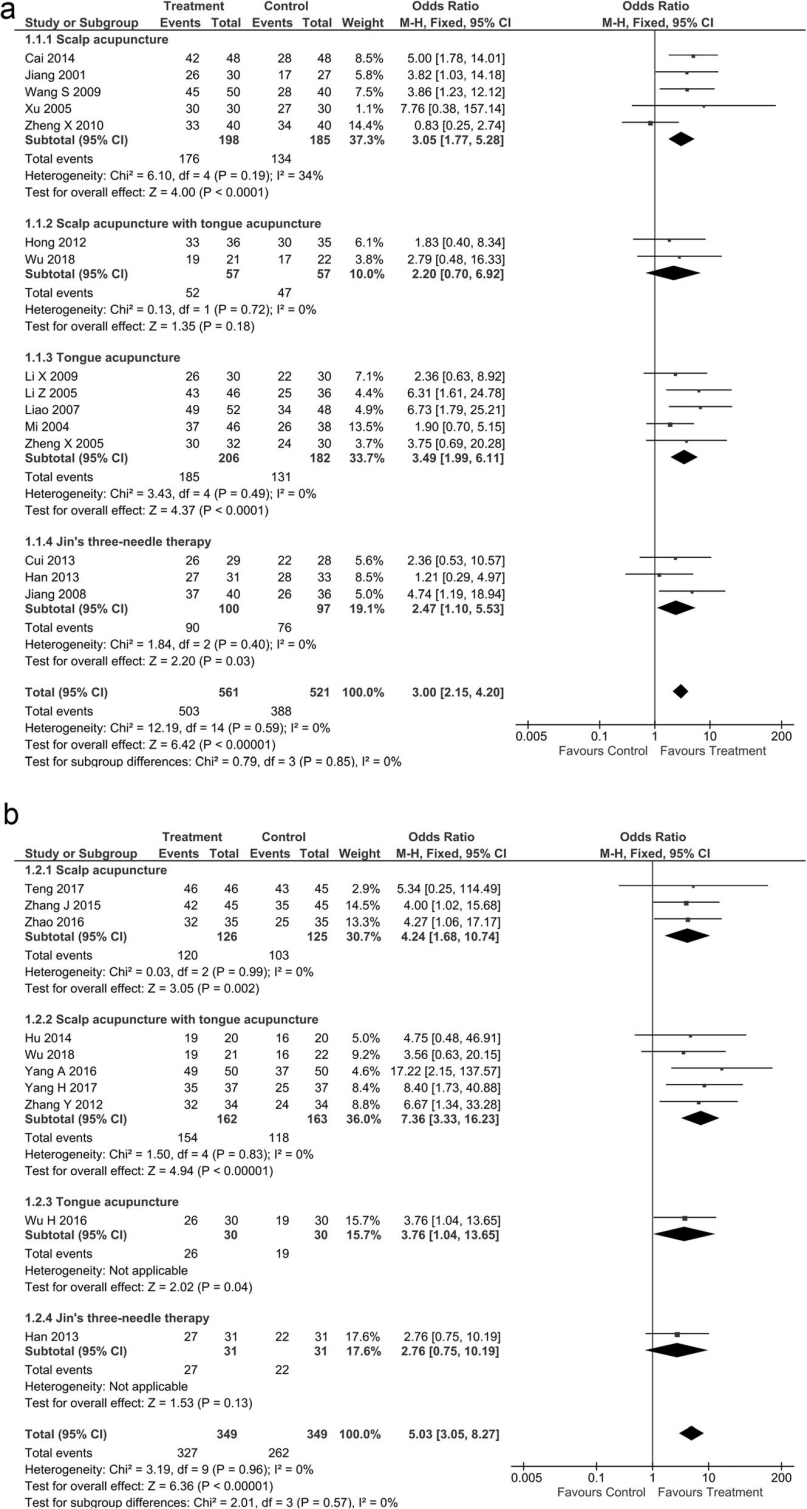
Forest graph showing the meta-analysis of odds ratios of the total effective rate in the (**a**) investigational group compared to the (**b**) control group

Total effective rate was reported by 29 studies with 1148 participants in investigational and 1108 in control groups. Compared to traditional acupuncture (TA), scalp acupuncture (OR 3.05 [95% CI: 1.77, 5.28]; *p*<0.00001), tongue acupuncture (OR 3.49 [1.99, 6.11]; *p*<0.00001), and Jin’s 3-needle therapy (OR 2.47 [1.10, 5.53]; *p* = 0.03 had significantly better total effective rate (Fig. 3a). Compared to rehabilitation training (RT), scalp acupuncture (OR 4.24 [95% CI: 1.68, 10.74]; *p* = 0.002) and scalp acupuncture with tongue acupuncture (OR 7.36 [3.33, 16.23]; *p*<0.00001) had significantly better total effective rate (Fig. 3b).

### The ABC score

**Fig. 4:**
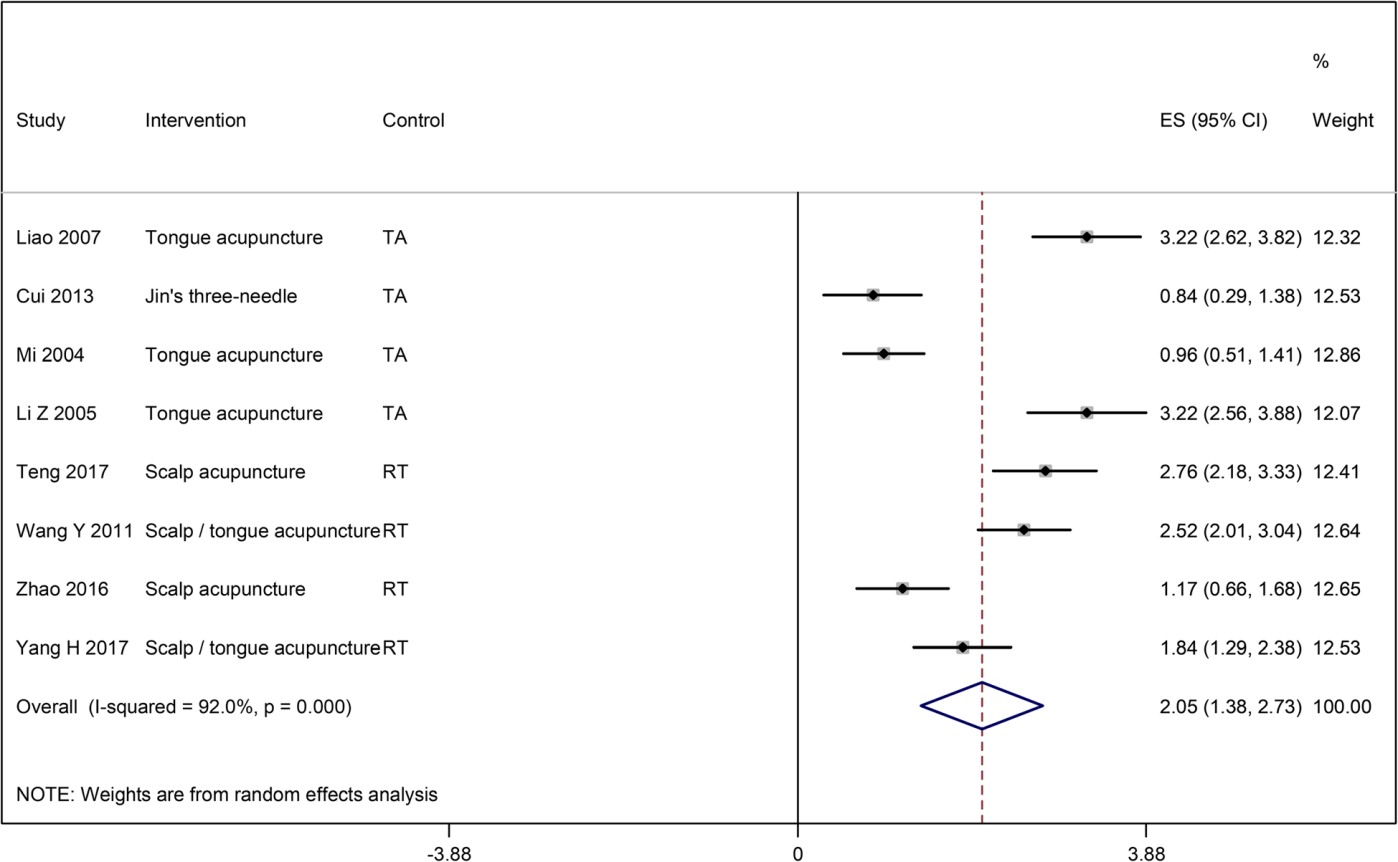
Forest plot of the ABC score in the investigational group compared to the control group

The ABC score was reported by 10 studies with 407 in investigational and 383 participants in control group. Investigational interventions improved the ABC score significantly more than control interventions (SMD: 2.05 [95% CI: 1.38, 2.73]; Fig. 4). In the subgroup analysis, we found that in comparison with control interventions, the ABC score improved significantly more with scalp acupuncture (SMD: 2.45 [95% CI: 0.84, 4.07]), tongue acupuncture (SMD 1.96 [95% CI: 0.41, 3.51]), Jin’s 3-needling (SMD: 0.84 [95% CI: 0.29, 1.38]), and scalp acupuncture with tongue acupuncture (SMD 2.53 [95% CI: 1.75, 3.32]) (Additional file 1: Figure S1).

### The comprehension score

The comprehension score was reported by 7 studies with 283 participants in investigational and 278 in control group. Overall, the comprehension score was significantly higher in investigational group compared to the control group (SMD: 0.54 [95% CI, 0.21, 0.86]; Additional file 1: Figure S2). In subgroup analysis, comprehension score was significantly higher after tongue acupuncture (SMD 0.85 [95% CI: 0.32, 1.38]) and scalp acupuncture with tongue acupuncture (SMD 0.50 [95% CI: 0.04, 0.97]) in comparison with control group Additional file 1: Figure S2). However, in other subgroups, there was no significant difference between the groups.

### The oral expression score

The oral expression score was reported by 6 studies with 254 participants in the investigational group and 250 in the control group. Compared to the control group, investigational group had significantly higher oral expression score (SMD 0.80 [95% CI: 0.44, 1.16]; Additional file 1: Figure S3). In the subgroup analysis, compared to control interventions, tongue acupuncture (SMD 0.89 [95% CI: 0.45, 1.32], scalp acupuncture (SMD 1.92 [95% CI: 1.31, 2.54]), Jin’s 3-needling (SMD 0.96 [95% CI: 0.48, 1.44]) and scalp acupuncture with tongue acupuncture (SMD 0.71 [95% CI: 0.38, 1.03] significantly improved the oral expression score (Additional file 1: Figure S3).

### The repetition score

The repetition score was reported by 7 studies with 267 participants in investigational group and 263 in control group. Investigational group had significantly higher repetition score compared to the control group (SMD 1.05 [95% CI: 0.64, 1.47]; Additional file 1: Figure S4). In the subgroup analysis, in comparison with control interventions, the repetition score was significantly higher after tongue acupuncture (SMD 1.95 [95% CI: 1.51, 2.39]), scalp acupuncture with tongue acupuncture (SMD 1.03 [95% CI: 0.34, 1.72]), and Jin’s 3-needling (SMD 0.78 [95% CI: 0.19,1.36]) (Additional file 1: Figure S4).

### The denomination score

The denomination score was reported by 6 studies with 231 participants in the investigational group and 228 in the control group. Compared to the control group, investigational group had significantly higher denomination score (SMD 0.82 [95% CI: 0.20, 1.44]; Additional file 1: Figure S5). In the subgroup analysis, in comparison with control interventions, tongue acupuncture (SMD 1.38 [95% CI: 0.42, 2.34**]**), scalp acupuncture with tongue acupuncture (SMD 1.16 [95% CI: 0.60, 1.71]), and Jin’s 3-needling (SMD 1.25 [95% CI: 0.88, 1.62] were associated with significantly higher denomination score (Additional file 1: Figure S5).

### The reading score

The reading score was reported by 4 studies with 147 participants in the investigational group and 147 in the control group. Investigational group had significantly higher reading score compared to the control group (SMD 0.90 [95% CI: 0.51, 1.30]; Additional file 1: Figure S6). In subgroup analysis, the reading score was significantly higher with scalp acupuncture (SMD 0.48 [95% CI 0.06, 0.90], tongue acupuncture (SMD 1.53 [95% CI: 0.95, 2.10], scalp acupuncture with tongue acupuncture (SMD 0.75 [95% CI: 0.28, 1.23]), and Jin’s 3-needling (SMD 1.08 [95% CI: 0.52, 1.64]) in comparison with control interventions (Additional file 1: Figure S6).

### The writing score

The writing score was reported by 4 studies with 147 participants in the investigational group and 147 in the control group. Compared to the control group, investigational group had significantly higher writing score after therapy (SMD 1.56 [95% CI: 0.62, 2.49]; Additional file 1: Figure S7). In subgroup analysis, the writing score was significantly higher in scalp acupuncture (SMD 1.20 [95% CI: 0.76, 1.65]), tongue acupuncture (SMD 3.72 [95% CI: 2.88, 4.57]), and scalp acupuncture with tongue acupuncture (SMD 1.36 [95% CI: 0.82, 1.91] in comparison with control interventions (Additional file 1: Figure S8). However, there was no statistically significant difference in the writing score after Jin’s 3-needling (SMD 0.29 [95% CI: − 0.23, 0.81] in comparison with control intervention (Additional file 1: Figure S7).

## Discussion

This meta-analysis found that compared to traditional acupuncture, scalp / tongue / Jin’s 3-needle acupuncture yield significantly better total effective rate, whereas compared to rehabilitation training, scalp acupuncture and scalp acupuncture with tongue acupuncture yield significantly better total effective rate. The ABC score also improved significantly more in scalp acupuncture, tongue acupuncture, Jin’s 3-needling, and scalp acupuncture with tongue acupuncture in comparison with traditional acupuncture or rehabilitation training. Moreover, comprehension, oral expression, repetition, denomination, reading, and writing also improved more with scalp, tongue and Jin’s 3-needle acupuncture, in general.

Previously, Zhang et al. [11] who conducted a meta-analysis of studies which used acupuncture for poststroke aphasia found that acupuncture provides benefits in swallowing (RR 1.61 [95% CI 0.73, 3.58]), neurological function (RR 6.55 [95% CI 1.89, 22.76]) and disability (RR 12.5 [95% CI 43.1,36.2]). Wu et al. [12] also conducted a meta-analysis of 38 trials and found an OR of 4.33 [95% CI 3.09, 6.08] in favor of acupuncture for providing improvement in poststroke rehabilitation but they concluded that quality of included studies was inadequate, and possibility of publication could not be ruled out.

In the present study, not only the total effective rate was better with scalp acupuncture, but it was also associated with significantly improved ABC score and its sub-scores. Scalp acupuncture stimulate different scalp zones to improve the reflexively of nervous tissue [8]. Litscher et al. [46] after monitoring cerebral oxygen saturation in 12 subjects during and after acupuncture have shown that cerebral oxygen saturation increased from 69.9% before to 70.3% during and 70.2% after acupuncture (*p* < 0.01). Studies in animals and humans have proven the effectiveness of scalp acupuncture in improving neurologic deficits by altering hormone levels in circulation and blood flow in the brain [47].

Tongue acupuncture was also associated with higher effectiveness rate and significantly improved ABC score and communication skills including comprehension, oral expression, repetition, denomination, reading and writing, in the present study. Tongue acupuncture targets acupoints on tongue for either single or repetitive stimulation [9]. Tongue acupuncture is found beneficial in improving symptoms in participants with chronic neurological conditions including autism, cerebral palsy, stroke, and drooling problems. Because tongue is an important part which is involved in many ingestion tasks and speech, it is rich in neural networks, vascular supply, and lymphatics. Thus, stimulation of key areas which link the vascularelymphatice neural networks by identifying acupoints to insert needles may trigger re-signaling of nerves and the potentiation of neurotransmission [48].

We have also found that Jin’s 3-needles acupuncture significantly improved ABC scores and communication skills. In the present study, total effective rate was statistically non-significantly higher, and the ABC score was significantly better in Jin’s 3-needles acupuncture group, besides oral expression, repetition, denomination, reading and writing improved significantly in Jin’s 3-needles acupuncture group. Jin’s 3 needles acupuncture is usually based on symptom such as slurred speech, difficulty swallowing, spastic paralysis, aphasia, etc. [10]. Several authors have reported that Jin’s 3-needle acupuncture was effective in improving cognitive and motor functions, hemiplegia and activities of daily living in stroke participants. Increase in cerebral blood flow coinciding with electrophysiological activities has been reported by many studies [49].

Although, the findings of the present study support the use of scalp, tongue, and Jin’s 3-needle acupuncture for post-stroke rehabilitation based on data obtained from 32 studies, many caveats are needed to be taken care of before clinical implications of this evidence could be reined. Quality of research is not adequate so far. There is less information available with regards to the follow-up of studies. There is also a need to use health-related quality of life tools to appraise participants for their outcomes with reasonable follow-up. If such a survey could be inculcated with a large sized randomized controlled trial, it could be the most useful.

In stroke rehabilitation there is essential and urgent need that patient should be involved a total complex rehabilitation work, which should be built according to most recent situation and symptoms of individual patients. Moving exercises, professional logopedics intervention are much needed, but daily gymnastic (active /passive) is necessary. Acupuncture is a valuable and less expensive additional link to have better output from rehabilitation process. The number of acupuncture clients is increasing and therefore this modality can be benefitted with research and feedback.

Some limitations of the present study should be considered while interpreting the results. These include: (i) only randomized controlled trails were used, (ii) variables with regards to previous disease condition and treatments were unavailable for many studies, (iv) some studies of low quality and with a low Jadad score were included, and (v) treatment time and protocols may have varied across the included studies.

## Conclusions

In this meta-analysis, we have found that, compared to traditional acupuncture and/or rehabilitation training, scalp acupuncture, tongue acupuncture, and Jin’ 3-needle acupuncture can better improve post-apoplectic aphasia as depicted by the total effective rate, the ABC score, and comprehension, oral expression, repetition, denomination, reading and writing scores. However, quality of the included studies was inadequate and therefore further high-quality studies with lager samples and longer follow-up times and with patient outcomes are necessary to verify the results presented herein. In future studies, researchers should also explore the efficacy and differences between scalp acupuncture, tongue acupuncture and Jin’s 3-needling in the treatment of postapoplectic aphasia.

## Supplementary information

**Additional file 1 **: **Table S1**. Characteristics of the included studies. **Figure S1.** Forest graph showing the subgroup analyses of the SMDs in ABC scores between investigational and control groups. **Figure S2.** Forest plot of the score of comprehension in the investigational group compared to the control group in the subgroup analysis. **Figure S3.** Forest plot of the score of oral expression in the investigational group compared to the control group in the subgroup analysis. **Figure S4.** Forest plot of the score of repetition in the investigational group compared to the control group in the subgroup analysis. **Figure S5.** Forest plot of the score of denomination in the investigational group compared to the control group in the subgroup analysis. **Figure S6.** Forest plot of the score of reading in the investigational group compared to the control group in the subgroup analysis. **Figure S7.** Forest plot of the score of writing in the investigational group compared to the control group in the subgroup analysis**. Appendix S1** (Literature search strategy)

## Data Availability

The datasets used and/or analysed during the current study are available from the corresponding author on reasonable request.

## Abbreviations

ABC: Aphasia Battery of Chinese
MD: Mean difference
OR: Odds ratio
RR: Relative risk

## References

[1] Donnan GA, Fisher M, Macleod M, Davis SM (2008). Stroke. Lancet.

[2] Murray CJ, Lopez AD (1997). Mortality by cause for eight regions of the world: global burden of disease study. Lancet.

[3] American Heart Association, 2009 Update at-a-Glance (2009). Heart Disease and Stroke Statistics.

[4] Feigin VL, Norrving B, Mensah GA (2017). Global burden of stroke. Circ Res.

[5] World Health Organization. Stroke: A global response is needed. https://www.who.int/bulletin/volumes/94/9/16-181636/en/. Accessed 1 Aug 2019.

[6] American Heart Association. Heart Disease and Stroke Statistics 2018 At-a-Glance. https://www.heart.org/-/media/data-import/downloadables/heart-disease-and-stroke-statistics-2018%2D%2D-at-a-glance-ucm_498848.pdf. Accessed 1 Aug 2019.

[7] Guzik A, Bushnell C (2017). Stroke epidemiology and risk factor management. Continuum (Minneap Minn).

[8] Allam H, ElDine NG, Helmy G (2008). Scalp acupuncture effect on language development in children with autism: a pilot study. J Altern Complement Med.

[9] Cai H, Ma B, Gao X, Gao H (2015). Tongue acupuncture in treatment of post-stroke dysphagia. Int J Clin Exp Med.

[10] Zhou Z, Zhuang L, Xu ZLiao M, Zhang B (2013). Effect of combined Jin’s 3-needle and rehabilitation care on post-stroke hemiplegia. J Acupunct Tuina Sci..

[11] Zhang X, Liu X, Kang D (2015). GRADE in systematic reviews of acupuncture for stroke rehabilitation: recommendations based on high-quality evidence. Sci Rep.

[12] Wu P, Mills E, Moher D, Seely D (2010). Acupuncture in poststroke rehabilitation: a systematic review and meta-analysis of randomized trials. Stroke.

[13] Ottawa P, Khadilkar A, Phillips K, Jean N, Lamothe C, Milne S (2006). Ottawa panel evidence-based clinical practice guidelines for post-stroke rehabilitation. Top Stroke Rehabil.

[14] Cai F, Gu W, Shi X (2014). Clinical observation of “Xingnao Kaiqiao” acupuncture therapy oil aphasia after cerebral infarction. Tianjin J Tradit Chin Med.

[15] Chen G, Yang Y, Ni F, Sang L (2018). Study of pestle needle therapy for speech rehabilitation in patients with post-stroke motor aphasia. Shang J Acu-mox.

[16] Cui S, Liu J, Wang S, Xu M, Lai C (2013). Clinical observations of tongue three-needle combined with temporal three-needle in stroke aphasia patients. JETCM.

[17] Gu S (2014). Clinical value analysis of acupuncture treatment for aphasia due to apoplexy. Guide China Med.

[18] Han DX, Zhang Y (2013). Clinical research on aphasia after stroke by Jin's three needles therapy combined with speech training. J Zhejiang Chin Med Univ.

[19] Hong Q, Wang S (2012). Clinical observation of Dongshi qi point combined with body acupuncture in treating aphasia after stroke. Beijing J Tradit Chin Med.

[20] Hu JL (2014). Clinical observation of acupuncture combined with language rehabilitation training in treatment of stroke aphasia. Yiyao Qianyan.

[21] Jiang GH, Li XL (2008). Observations of the efficacy of Jin’s tongue three needles in treating apoplectic motor aphasia. Shanghai J Acupunct Moxibustion.

[22] Jiang GH, Li YH, Chen ZH (2001). Clinical observation of surround needling under CT location for treatment of aphasia due to apoplexy. Chin Acupunct Moxibustion.

[23] Li G, Zhao X (2011). Clinical study on combined acupuncture and speech rehabilitation in treating postapoplectic aphasia. J Acupunct Tuina Sci..

[24] Li L, Yue Z (2011). Blood-letting therapy combined with speech rehabilitation in the treatment of motor aphasia after stroke. JCAM.

[25] Li X, Cai J, Jinag G (2009). Observation on clinical effect of tongue triple acupuncture in the treatment of 30 aphasia after stroke patients. JCAM.

[26] Li Z, Fu W (2005). Treatment of anandia after apoplexy by tongue acupuncture therapy: a clinical observation of 46 cases. New J Tradit Chin Med.

[27] Liao J (2007). The treatment of bloodletting in pointes of Jinjin and Yuye on 50 aphemia due to stroke patients. JCAM.

[28] Luo W, Huang H, Zhu J (2008). Acupuncture combined with language training for treatment of motor aphasia caused by ischemic apoplexy. World J Acu-moxi.

[29] Ma D, He R (2010). The curative effect observation of acupuncture with rehabilitation training on treating palsy aphasia. China J Chin Med.

[30] Mi J, Zhu X (2004). ClinicaI observations on the treatment of 46 apoplectic aphasia patients by tongue acupuncture as a main method. Shanghai J Acu-mox.

[31] Qin X, Liu L (2011). Clinical observation of acupuncture combined with rehabilitation in the treatment of stroke sequela. Today Nurse.

[32] Teng YY (2017). Clinical observation of scalp acupuncture plus speech rehabilitation for Broca’s aphasia after cerebral stroke. J Acupunct Tuina Sci.

[33] Tian L, Hu R, Lou T (2015). Effect of acupuncture plus language function training on language function in patients with post-stroke aphasia. Shanghai J Acu-mox.

[34] Wang S, Yang C (2001). Observation of 50 cases of aphasia after stroke treated by scalp acupuncture. J New Chin Med.

[35] Wang Y, Li A, Wang H, Du K, Du Y (2011). Acupuncture combined with language training in the treatment of aphasia after stroke. JCAM.

[36] Wu H, Xu G, Zeng K (2016). Clinical study of speech training with taiji acupuncture on patients with aphasia after wind stroke. World Chin Med.

[37] Wu K, Guo X, Wu Z (2018). Clinical observation of tongue three-needle combined with scalp acupuncture in the treatment of stroke aphasia. JCAM.

[38] Xu Y, Li Q, Hao Y (2005). Observation on the efficacy of acupuncture plus rehabilitation composite treatment for apoplectic aphasia. Shanghai J Acu-mos.

[39] Yang A (2016). Clinical study on treatment of sequela of apoplectic with hemiplegia with acupuncture and rehabilitation training. JCAM.

[40] Yang HB, Hou BG, Zhang ZL, Sun JH, Yang LM (2017). Research on the effect of acupuncture combined with language rehabilitation training in treatment of stroke aphasia. Home Med.

[41] Zhang J (2015). The feasibility study on acupuncture combined with language training in the treatment of aphasia after stroke. Psychol Doct.

[42] Zhang Y, Yu Z, Zhang Y, Zhao X, Lin C (2012). Therapeutic observation on acupuncture and rehabilitation in treating motor aphasia after cerebral apoplexy. Shanghai J Acu-mox.

[43] Zhao C, Tong Z, Hunag C, You Y (2016). Research on the effect of acupuncture combined with language rehabilitation training in treatment of stroke aphasia. Pract Clin J Integr Tradit Chin West Med.

[44] Zheng X, Zhu Q, Lin Y, Liu T (2010). Effect of blood-letting puncture on endothelin-1 and treatment for stroke aphasia. Hubei J TCM.

[45] Zheng XB (2005). 32 cases of sports aphasia treated by blood letting puncture at the base of tongue. Shanghai J Acu-mox.

[46] Litscher G, Schwarz G (1998). Effects of acupuncture on oxygenation of cerebral tissue. Neurol Res.

[47] Xiang L, Wang H, Li Z (1996). TCD observation on cerebral blood flow dynamics: inference of cerebral palsy with scalp therapy. Acupunct Res.

[48] Wong VC, Sun JG (2010). Randomized controlled trial of acupuncture versus sham acupuncture in autism spectrum disorder. J Altern Complement Med.

[49] Yang X, Yu H, Zhang T, Luo X, Ding L, Chen B, Li D, Huang X, Guo X, Jia J. The effects of Jin’s 3-needle acupuncture therapy on EEG alpha rhythm of stroke participants. Top Stroke Rehabil. 2018;25(7):1–5. 10.1080/10749357.2018.1484680 30599806

