# Supplementary material for: Efficacy of acupuncture in the management of post-apoplectic aphasia: a systematic review and meta-analysis of randomized controlled trials
Source: BMC Complement Altern Med. 2019 Oct 25;19:282. doi: 10.1186/s12906-019-2687-1 (PMC6815030; doi:10.1186/s12906-019-2687-1)
Supplement: Supplementary file 1 — Additional file 1 : Table S1. Characteristics of the included studies. Figure S1. Forest graph showing the subgroup analyses of the SMDs in ABC scores between investigational and control groups. Figure S2. Forest plot of the score of comprehension in the investigational group compared to the control group in the subgroup analysis. Figure S3. Forest plot of the score of oral expression in the investigational group compared to the control group in the subgroup analysis. Figure S4. Forest plot of the score of repetition in the investigational group compared to the control group in the subgroup analysis. Figure S5. Forest plot of the score of denomination in the investigational group compared to the control group in the subgroup analysis. Figure S6. Forest plot of the score of reading in the investigational group compared to the control group in the subgroup analysis. Figure S7. Forest plot of the score of writing in the investigational group compared to the control group in the subgroup analysis. Appendix S1 (Literature search strategy) [file 12906_2019_2687_MOESM1_ESM.docx]

**Additional file 1**

| Table S1. Characteristics of the included studies | | | | | | | | | |
| --- | --- | --- | --- | --- | --- | --- | --- | --- | --- |
| Study | No. of patients | | Gender | | Age | | Therapy | | Jadad  score |
|  | I | C | I | C | I | C | I | C |  |
| Cai 2014 | 48 | 48 | 25M | 22M | 58.4 | 57.7 | Scalp acupuncture | TA | 3 |
| Chen 2018 | 36 | 36 | 18M | 18M | 63 | 64 | TA + RT | RT | 3 |
| Cui 2013 | 29 | 28 | 16M | 18M | 61.48 | 62.18 | Jin's Three-needling | TA | 2 |
| Gu 2014 | 36 | 36 | 23M | 22M | 61.9 | 61.7 | TA + RT | RT | 3 |
| Han 2013 | 31 | 33 | 16M | 17M | 63.4 | 62.7 | Jin's Three-needling + RT | TA + RT | 3 |
| Han 2013 | 31 | 31 | 16M | 17M | 63.4 | 63.6 | Jin's Three-needling + RT | RT | 3 |
| Hong 2012 | 36 | 35 | - | - | - | - | Scalp + tongue acupuncture | TA | 2 |
| Hu 2014 | 20 | 20 | 12M | 13M | 65.3 | 60.2 | Tongue acupuncture + RT | RT | 2 |
| Jiang 2001 | 30 | 27 | 18M | 17M | - | - | Scalp acupuncture | TA | 2 |
| Jiang 2008 | 40 | 36 | 23M | 23M | 41.6 | 39.6 | Jin's Three-needling | TA | 2 |
| Li G 2011 | 30 | 30 | 12M | 14M | 57.6 | 58.3 | TA + RT | RT | 3 |
| Li L 2011 | 36 | 36 | 21M | 19M | 65.16 | 62.45 | TA + RT | RT | 3 |
| Li X 2009 | 30 | 30 | 18M | 20M | 61.5 | 59.2 | Tongue acupuncture | TA | 2 |
| Li Z 2005 | 46 | 36 | 28M | 24M | 61.5 | 59.2 | Tongue acupuncture | TA | 2 |
| Liao 2007 | 52 | 48 | 38M | 32M | 61.5 | 59.2 | Tongue acupuncture + TA | TA | 2 |
| Luo 2008 | 30 | 30 | 19M | 16M | 60 | 62.2 | Tongue acupuncture + RT | RT | 2 |
| Ma 2010 | 30 | 30 | - | - | - | - | TA + RT | RT | 2 |
| Mi 2004 | 46 | 38 | 26M | 20M | 33-75 | 40-73 | Tongue acupuncture + TA | TA | 2 |
| Qin 2011 | 40 | 40 | - | - | - | - | TA + RT | RT | 3 |
| Teng 2017 | 46 | 45 | 24M | 25M | 56.3 | 57.6 | Scalp acupuncture | RT | 2 |
| Tian 2015 | 30 | 30 | 23M | 21M | 60 | 60 | TA + RT | RT | 2 |
| Tian 2015 | 30 | 30 | 23M | 21M | 60 | 60 | TA | RT | 2 |
| Wang S 2009 | 50 | 40 | - | - | - | - | Scalp acupuncture | TA | 2 |
| Wang Y 2011 | 52 | 52 | 27M | 28M | 66.2 | 65.9 | Scalp acupuncture + TA + RT | RT | 3 |
| Wu K 2018 | 21 | 22 | 12M | 15M | 62.4 | 63.8 | Scalp + tongue acupuncture | TA | 3 |
| Wu K 2018 | 21 | 22 | 12M | 13M | 62.4 | 61.7 | Scalp + tongue acupuncture | RT | 3 |
| Wu H 2016 | 30 | 30 | - | - | - | - | Tongue acupuncture | RT | 2 |
| Wu H 2016 | 30 | 30 | - | - | - | - | TA | RT | 2 |
| Xu 2005 | 30 | 30 | - | - | - | - | Scalp acupuncture + TA + RT | TA | 2 |
| Yang A 2016 | 50 | 50 | 28M | 30M | 68.56 | 69.87 | Scalp acupuncture + TA | RT | 3 |
| Yang H 2017 | 37 | 37 | 20M | 19M | 58.7 | 58.3 | Scalp acupuncture + RT | RT | 2 |
| Zhang J 2015 | 45 | 45 | 24M | 23M | 58.42 | 58.69 | Scalp acupuncture + TA | RT | 3 |
| Zhang Y 2012 | 34 | 34 | 20M | 18M | 52 | 55 | Scalp acupuncture + TA | RT | 2 |
| Zhao 2016 | 35 | 35 | 23M | 25M | 51.16 | 50.48 | Scalp acupuncture + RT | RT | 2 |
| Zheng 2005 | 32 | 30 | 20M | 17M | 62.5 | 63.8 | Tongue acupuncture | TA | 2 |
| Zheng 2010 | 40 | 40 | - | - | - | - | Scalp acupuncture | TA | 3 |

**Abbreviations: C, Control group; I, intervention group; RT, rehabilitation training; TA, traditional acupuncture.**

**
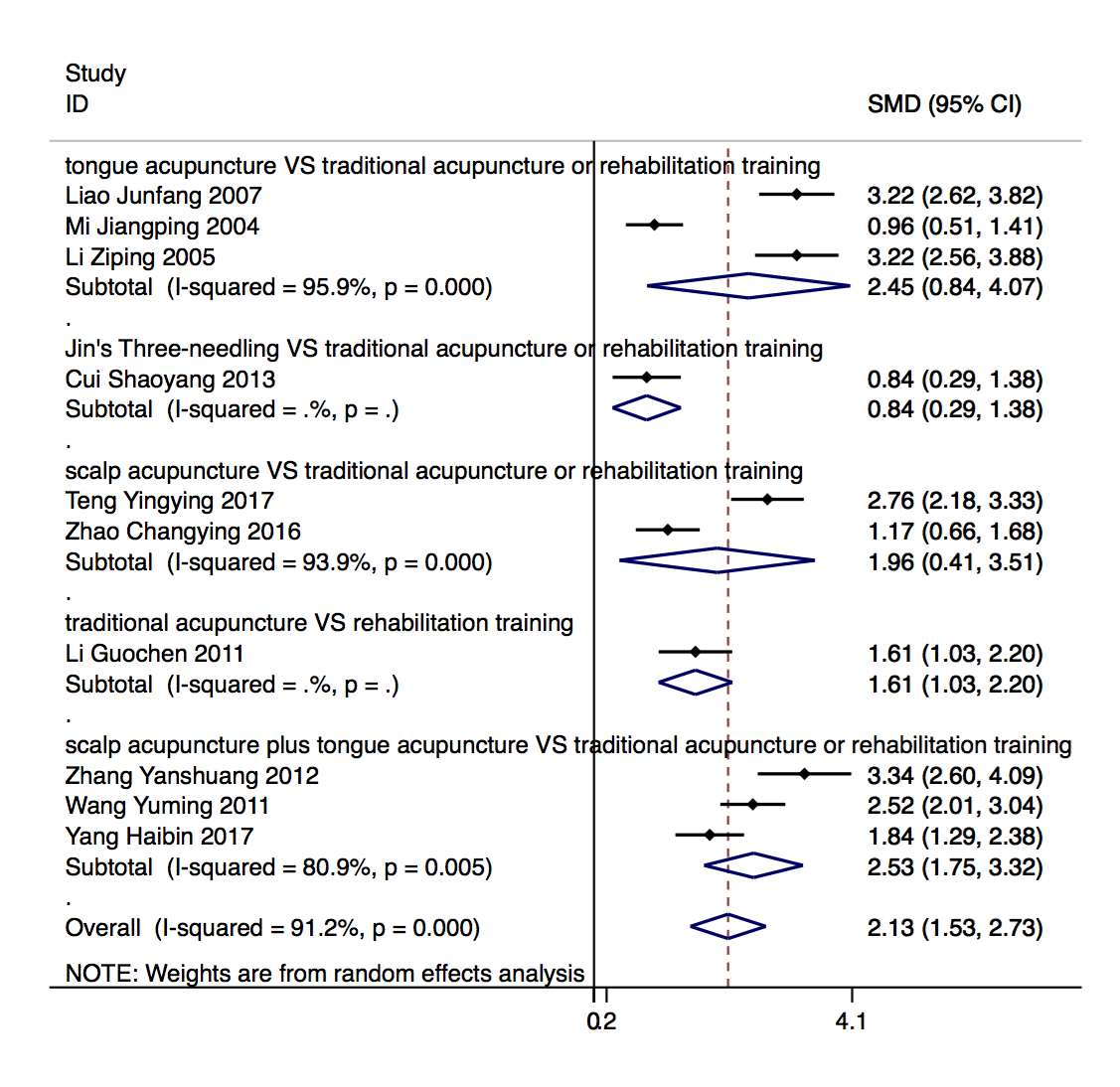
**

**Figure S1. Forest graph showing the subgroup analyses of the SMDs in ABC scores between investigational and control groups.**

**
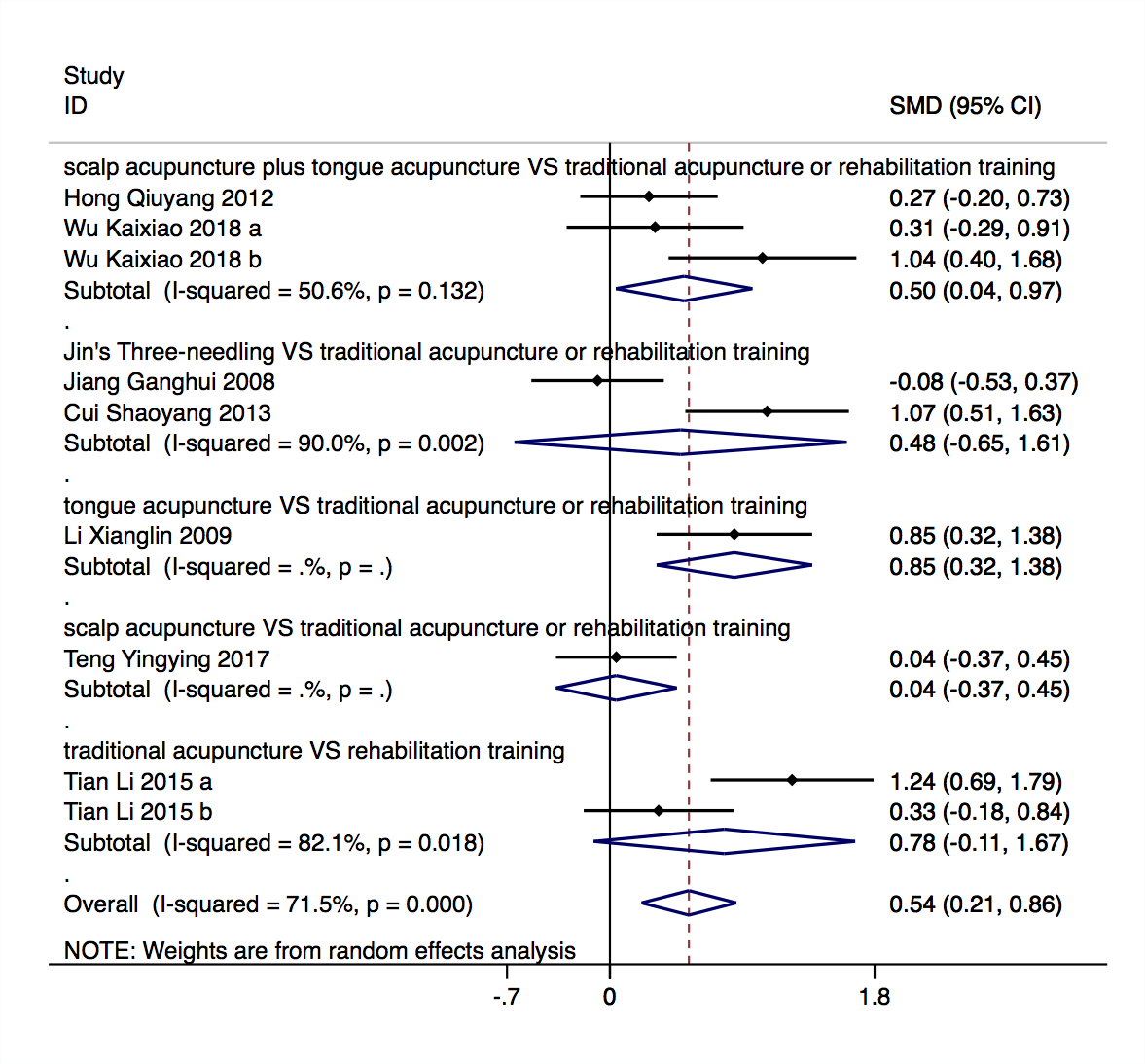
**

**Figure S2.** **Forest plot of the score of comprehension in the investigational group compared to the control group in the subgroup analysis.**

**
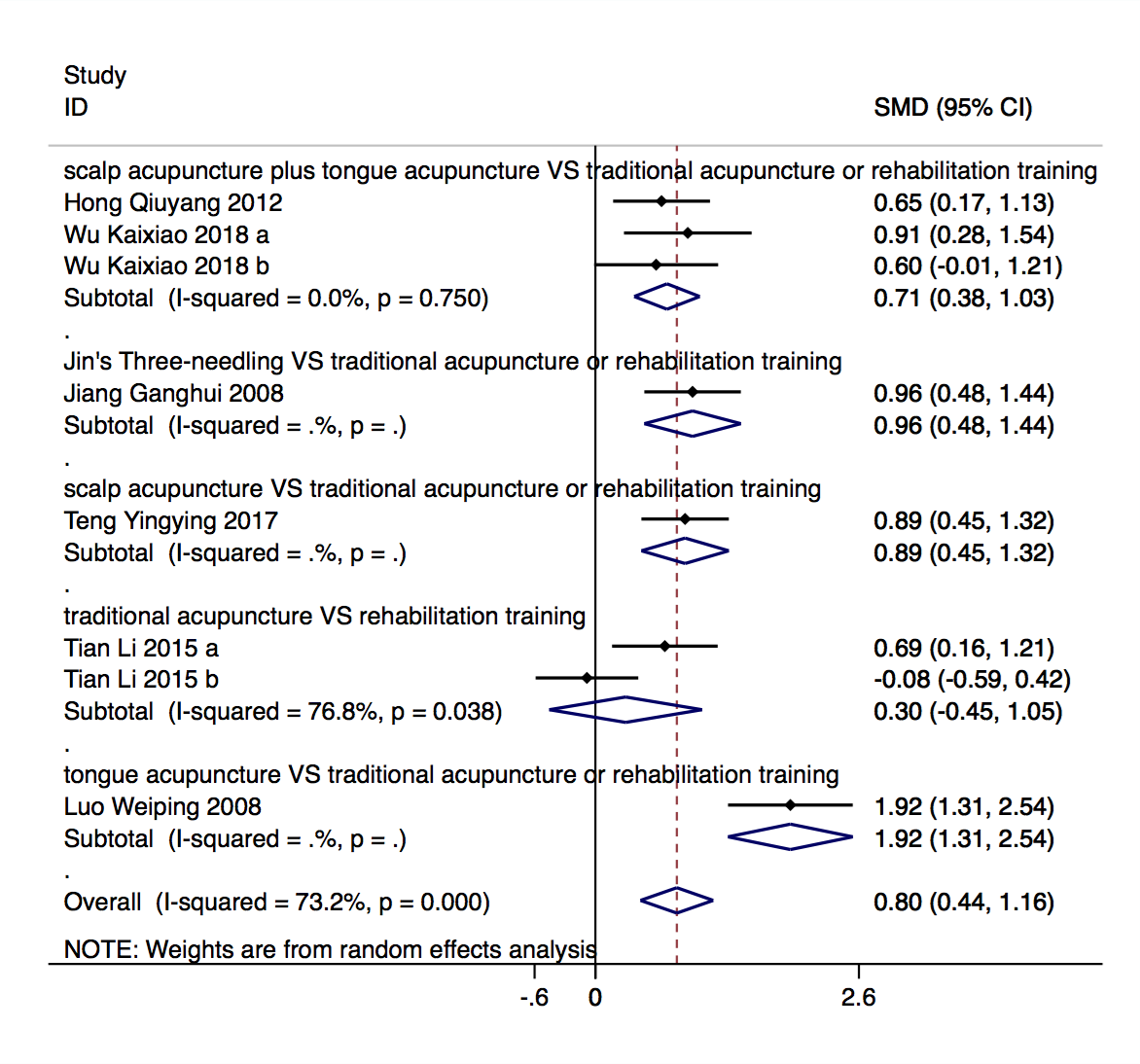
**

**Figure S3.** **Forest plot of the score of oral expression in the investigational group compared to the control group in the subgroup analysis.**

**
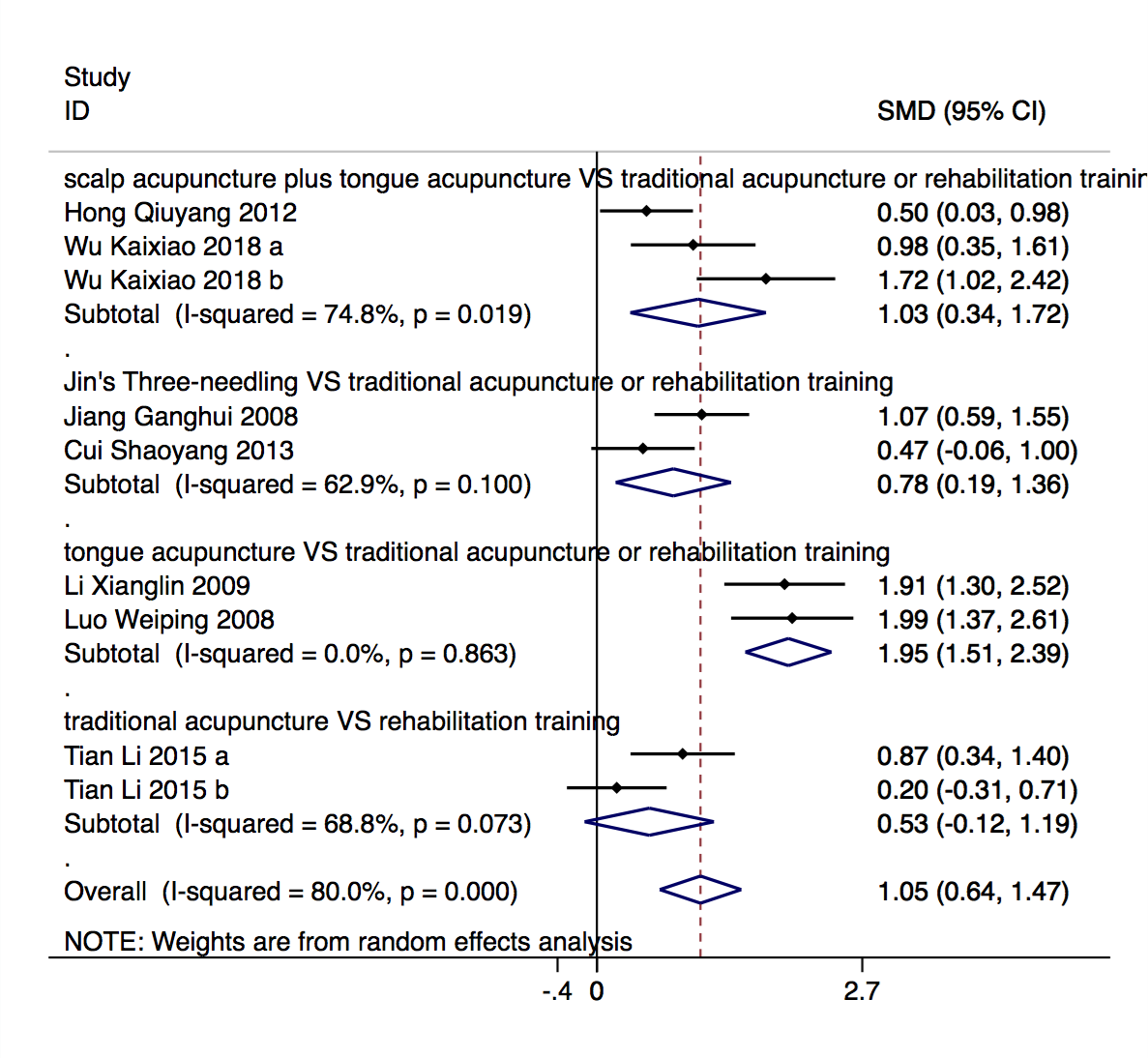
**

**Figure S4.** **Forest plot of the score of repetition in the investigational group compared to the control group in the subgroup analysis.**

**
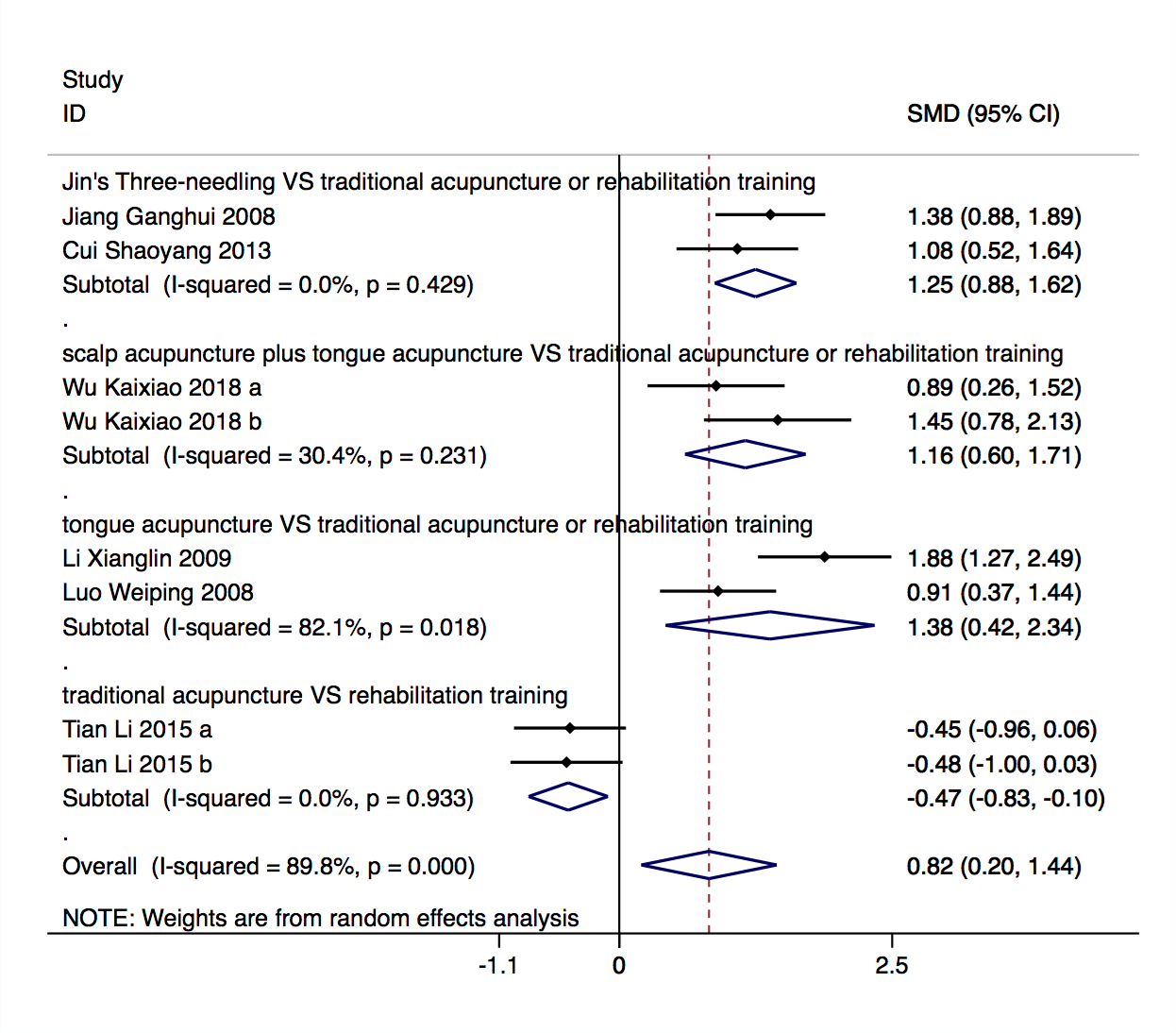
**

**Figure S5. Forest plot of the score of denomination in the investigational group compared to the control group in the subgroup analysis.**

**
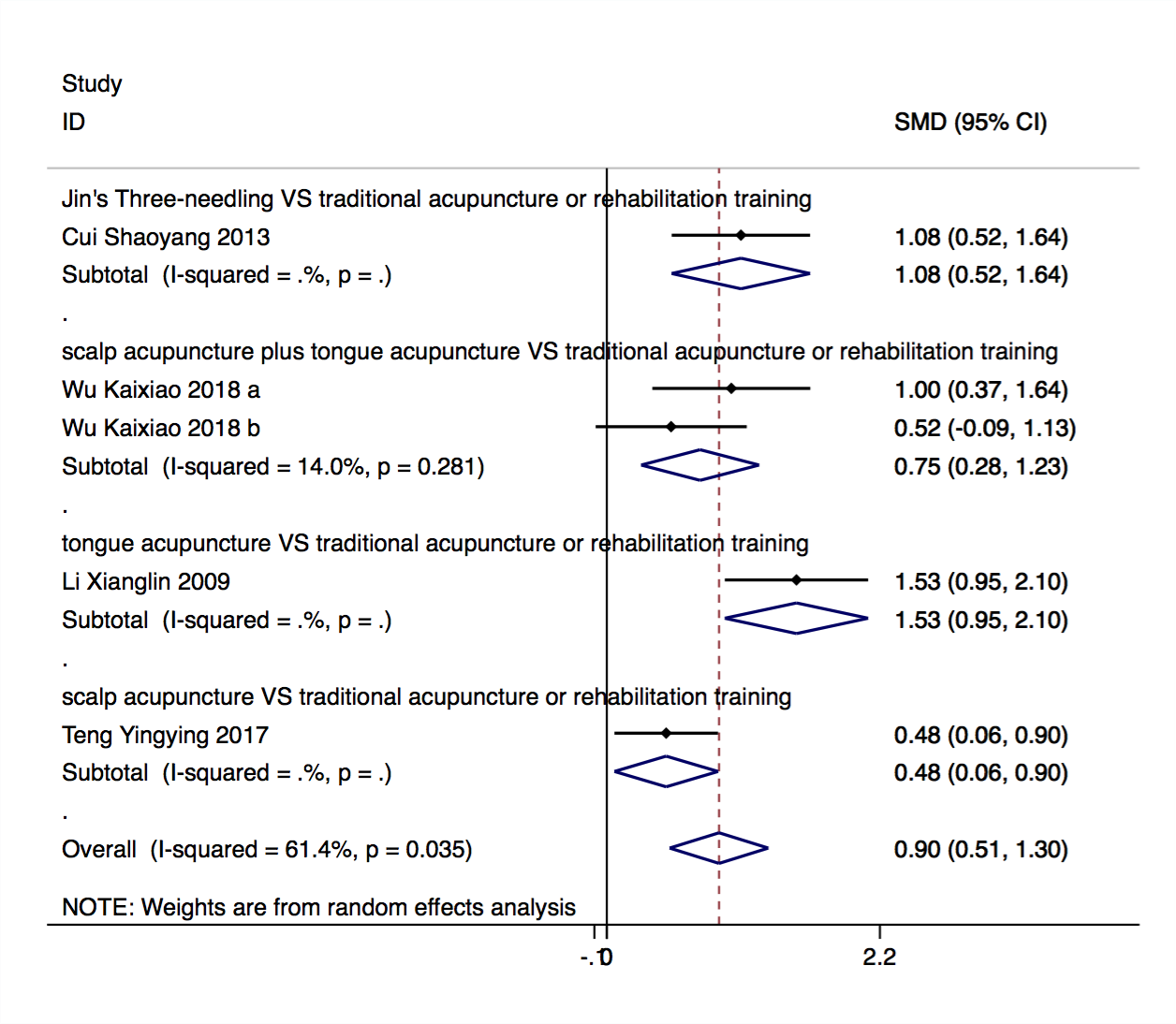
**

**Figure S6.** **Forest plot of the score of reading in the investigational group compared to the control group in the subgroup analysis.**

**
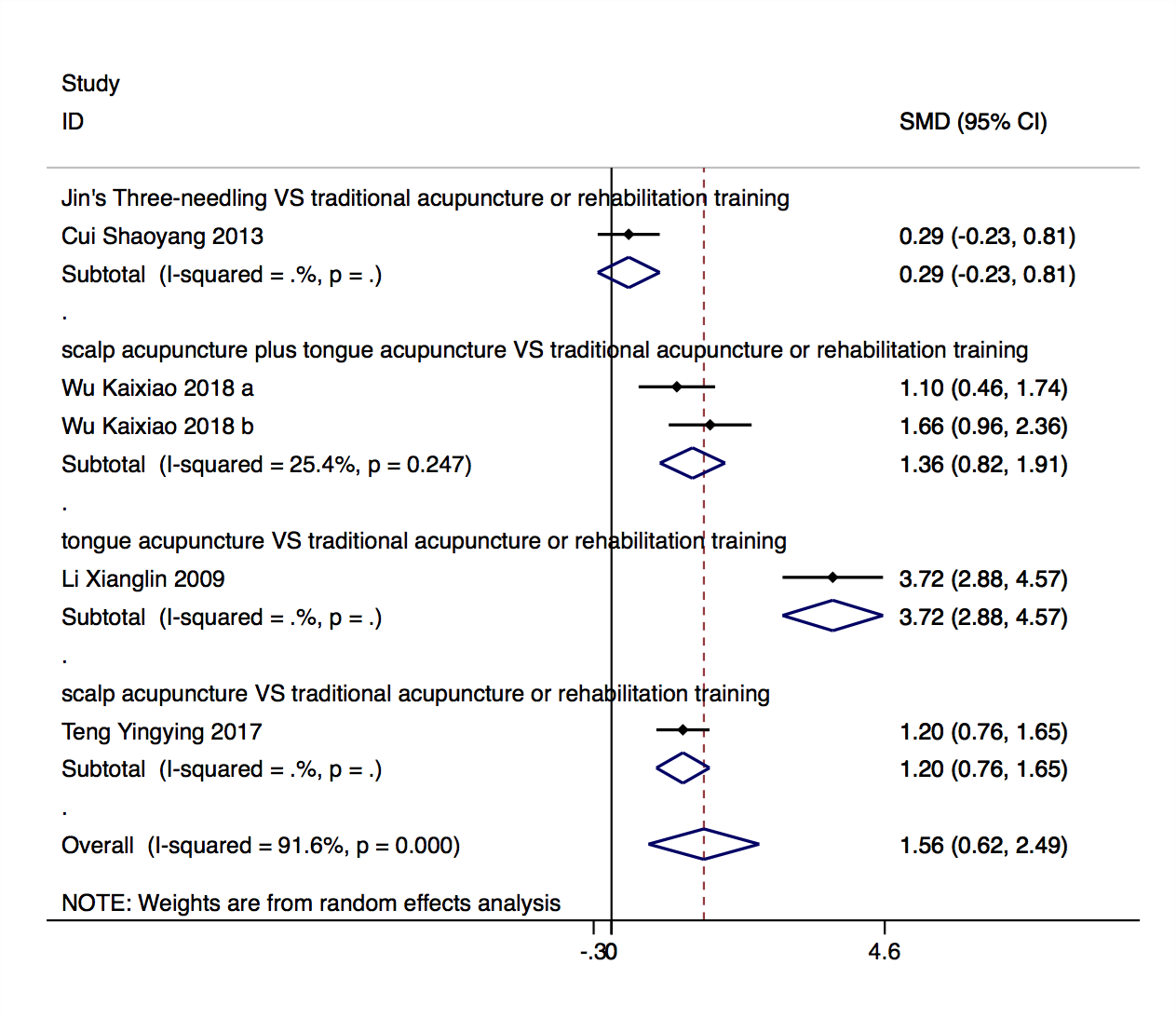
**

**Figure S7.** **Forest plot of the score of writing in the investigational group compared to the control group in the subgroup analysis.**

**Appendix S1 (Literature search strategy)**

("stroke"[MeSH Terms] OR "stroke"[All Fields]) AND ("aphasia"[MeSH Terms] OR "aphasia"[All Fields]) AND ("acupuncture"[MeSH Terms] OR "acupuncture"[All Fields] OR "acupuncture therapy"[MeSH Terms] OR ("acupuncture"[All Fields] AND "therapy"[All Fields]) OR "acupuncture therapy"[All Fields])

("stroke"[MeSH Terms] OR "stroke"[All Fields]) AND ("acupuncture"[MeSH Terms] OR "acupuncture"[All Fields] OR "acupuncture therapy"[MeSH Terms] OR ("acupuncture"[All Fields] AND "therapy"[All Fields]) OR "acupuncture therapy"[All Fields]) AND ("aphasia"[MeSH Terms] OR "aphasia"[All Fields]) AND Battery[All Fields] AND ("asian continental ancestry group"[MeSH Terms] OR ("asian"[All Fields] AND "continental"[All Fields] AND "ancestry"[All Fields] AND "group"[All Fields]) OR "asian continental ancestry group"[All Fields] OR "chinese"[All Fields])

("stroke"[MeSH Terms] OR "stroke"[All Fields]) AND ("acupuncture"[MeSH Terms] OR "acupuncture"[All Fields] OR "acupuncture therapy"[MeSH Terms] OR ("acupuncture"[All Fields] AND "therapy"[All Fields]) OR "acupuncture therapy"[All Fields]) AND ("aphasia"[MeSH Terms] OR "aphasia"[All Fields]) AND ("randomized controlled trial"[Publication Type] OR "randomized controlled trials as topic"[MeSH Terms] OR "randomized controlled trial"[All Fields] OR "randomised controlled trial"[All Fields])

effects[Title] AND Jin's[Title] AND three-needle[Title] AND acupuncture[Title] AND therapy[Title] AND EEG[Title] AND alpha[Title] AND rhythm[Title] AND stroke[Title] AND patients[Title]

post-stroke[All Fields] AND ("acupuncture"[MeSH Terms] OR "acupuncture"[All Fields] OR "acupuncture therapy"[MeSH Terms] OR ("acupuncture"[All Fields] AND "therapy"[All Fields]) OR "acupuncture therapy"[All Fields]) AND efficacy[All Fields]

post-stroke[All Fields] AND ("acupuncture"[MeSH Terms] OR "acupuncture"[All Fields] OR "acupuncture therapy"[MeSH Terms] OR ("acupuncture"[All Fields] AND "therapy"[All Fields]) OR "acupuncture therapy"[All Fields]) AND ("aphasia"[MeSH Terms] OR "aphasia"[All Fields])

post-stroke[All Fields] AND ("tongue"[MeSH Terms] OR "tongue"[All Fields]) AND ("acupuncture"[MeSH Terms] OR "acupuncture"[All Fields] OR "acupuncture therapy"[MeSH Terms] OR ("acupuncture"[All Fields] AND "therapy"[All Fields]) OR "acupuncture therapy"[All Fields]) AND ("aphasia"[MeSH Terms] OR "aphasia"[All Fields])

post-stroke[All Fields] AND ("scalp"[MeSH Terms] OR "scalp"[All Fields]) AND ("acupuncture"[MeSH Terms] OR "acupuncture"[All Fields] OR "acupuncture therapy"[MeSH Terms] OR ("acupuncture"[All Fields] AND "therapy"[All Fields]) OR "acupuncture therapy"[All Fields]) AND ("aphasia"[MeSH Terms] OR "aphasia"[All Fields])

post-stroke[All Fields] AND ("scalp"[MeSH Terms] OR "scalp"[All Fields]) AND ("acupuncture"[MeSH Terms] OR "acupuncture"[All Fields] OR "acupuncture therapy"[MeSH Terms] OR ("acupuncture"[All Fields] AND "therapy"[All Fields]) OR "acupuncture therapy"[All Fields]) AND ("rehabilitation"[Subheading] OR "rehabilitation"[All Fields] OR "rehabilitation"[MeSH Terms])

post-stroke[All Fields] AND ("tongue"[MeSH Terms] OR "tongue"[All Fields]) AND ("acupuncture"[MeSH Terms] OR "acupuncture"[All Fields] OR "acupuncture therapy"[MeSH Terms] OR ("acupuncture"[All Fields] AND "therapy"[All Fields]) OR "acupuncture therapy"[All Fields]) AND ("rehabilitation"[Subheading] OR "rehabilitation"[All Fields] OR "rehabilitation"[MeSH Terms])

post-stroke[All Fields] AND jin's[All Fields] AND three-needle[All Fields] AND ("rehabilitation"[Subheading] OR "rehabilitation"[All Fields] OR "rehabilitation"[MeSH Terms])

post-stroke[All Fields] AND jin's[All Fields] AND three-needle[All Fields] AND efficacy[All Fields]
